# Algorithmic Identification of Multiple Sclerosis in the SAIL databank

**DOI:** 10.23889/ijpds.v9i5.2538

**Published:** 2024-09-18

**Authors:** Eileen Mitchell, Siobhán Murphy, Dermot O'Reilly, Michael O’Donnelly

**Affiliations:** 1 Queen's University

## Background

Multiple Sclerosis (MS) is a challenging disease to identify within large repositories of healthcare data. Diagnosis can be protracted requiring many modalities that may not form part of the repository data collection. Accurate case finding would have multiple applications for studying epidemiology.

## Aim

Develop a case finding algorithm within the Secure Anonymised Information Linkage (SAIL) Databank that can reliably elicit Multiple Sclerosis.

## Method

Utilising a cross sectional cohort study we used multiple datasets within SAIL; General Practice, Inpatient, Outpatient and Office of National Statistics to develop our case finding algorithm based on the coding nomenclatures and timing within these datasets. The results of this algorithm were then tested against two patient data sets: The UK MS Register (n=836), consisting of mostly self confirmed disease and a clinical cohort from South West Wales (n=713).

## Results

From 4,757,428 records, the algorithm identified 6,194 cases of MS within Wales on 31st December 2020 (prevalence 221.65 [95%CI 216.17–227.24] per 100,000). Case finding sensitivity and specificity was 96.8% and 99.9% for the clinically validated population-based cohort and sensitivity was 96.7% from the self-declared registry population.

## Conclusion

We successfully identified MS cases within Wales and verified this within two independent data sets.

